# Supplementary material for: Humoral and Cellular Immune Responses Against SARS-CoV-2 Following COVID-19 Vaccination in Older Adults: A Systematic Review
Source: Vaccines (Basel). 2025 Aug 12;13(8):852. doi: 10.3390/vaccines13080852 (PMC12389793; doi:10.3390/vaccines13080852)

Table S1. Methodological appendix.

Variable	Description of Heterogeneity
**Type of vaccine used**	The studies included different technological platforms, such as mRNA vaccines (Pfizer-BioNTech, Moderna), viral vector vaccines (Oxford-AstraZeneca), and inactivated vaccines (CoronaVac). These differences may directly influence the magnitude and type of cellular immune response induced.
**Population health status**	Some studies enrolled healthy older adults, while others included individuals with multiple comorbidities such as diabetes, chronic obstructive pulmonary disease, cardiovascular disease, chronic kidney disease, neurological conditions, autoimmune disorders, or immunosuppression. These conditions may modulate the immune response and affect comparability across studies.
**Participant age**	Although all studies focused on older adults, the age ranges varied significantly (from > 60 to > 70 years), potentially affecting baseline immune function and the ability to mount a cellular immune response.
**Number of vaccine doses**	Differences were observed in whether studies assessed immune responses after the second, third, or even fourth vaccine dose, introducing variability in the magnitude of reported cellular immunity.
**Timing of post-vaccination assessment**	Cellular immunity was evaluated at different time points following vaccination (e.g., from a few days to several weeks), potentially reflecting distinct phases of the immune response.
**Method used to assess cellular immunity**	A variety of techniques were used, including ELISpot, FluoroSpot, standard or high-dimensional flow cytometry, QuantiFERON, and AIM assays. These methods differ in sensitivity, specificity, and the types of T cells measured (e.g., CD4+, CD8+, IFN-γ-producing cells).
**Study design**	While most studies were cohort-based, some were randomized controlled trials, introducing variability in methodological rigor and risk of bias.

Table S2. Characterization of data from the included studies.

Author/ Year	Vaccine type	Technology	Population health status	Population characteristics	Measuremnt timing	Test or assay to evaluate CIR	Test or assay to evaluate HIR	Outcome measures
Tut et al. 2021	Pfizer-BioNTech<br>Oxford-AstraZeneca	mRNA<br>Viral vector	Healthy older adults	Older adults with one dose of the vaccine	6 days<br>52 days	Human IFN-γ ELISpotPRO	V-PLEX SARS-CoV-2 Panel 2	CIR<br>HIR
Costa et al. 2022	CoronaVac	Inactivated	Healthy older adults	Vaccinated older adults	14 days	AIM/flow cytometry	Electrochemiluminescence multiplex serology assay	CIR<br>HIR
Roouers et al. 2022	Pfizer-BioNTech<br>Moderna	mRNA	Healthy older adults	Older adults with two doses of the vaccine	28 days	ICS<br>Multicolored flow cytometry	ELISpot<br>Flow Cytometry<br>Pseudovirus neutralization	CIR<br>HIR
Chaiwong et al. 2023	CoronaVac<br>Oxford-AstraZeneca	Inactivated<br>Viral vector	Older adults with COPD	Vaccinated older adults	4 weeks	BD FACSCelestaTM flow cytometer	cPass SARS-CoV-2	CIR<br>HIR
Dalla et al. 2023	Pfizer-BioNTech	mRNA	Healthy older adults	Older adults with two doses of the vaccine	6 months	ELISpot	ELISA<br>Competitive ELISA for Nab	CIR<br>HIR
Dudley et al. 2023	Pfizer-BioNTech<br>Moderna	mRNA	Older adults with and without RA	Older adults with one dose of the vaccine	2 to 24 weeks	ELISPOT	Bead-multiplex immunoassay<br>Pseudovirus neutralization assay	CIR<br>HIR
Saiag et al. 2023	Pfizer-BioNTech	mRNA	Healthy older adults	Older adults with three doses of the vaccine	2 months	SARS-CoV-2 T-Cell Analysis Kits for human PBMCs	ADVIA Centaur SARS-CoV-2 IgG<br>CMIA	CIR<br>HIR
Bredholt et al.2024	Pfizer–BioNTech<br>Moderna	mRNA	Healthy older adults	Older adults with two doses of the vaccine	3 weeks to 18 months	FluoroSpot<br>ELISpot	ELISA<br>Microneutralization	CIR<br>HIR
Kometani et al. 2024	Pfizer-BioNTech	mRNA	Healthy older adults	Vaccinated older adults	3 to 6 months	AIM	Surrogate Virus Neutralization Assay<br>Flow cytometry	CIR<br>HIR

Niyomnaitham et al. 2024	Pfizer-BioNTech Oxford-AstraZeneca	mRNA Viral vector	Frailty older adults	Older adults with two doses of the vaccine	2 to 17 weeks	ELISpot	CMIA Neutralization assay	CIR HIR
Segato et al. 2024	Pfizer-BioNTech	mRNA	Healthy older adults	Older adults with three doses of the vaccine	1 month	ELISpot AIM	ELISA	CIR HIR
Vanda et al. 2024	Pfizer-BioNTech	mRNA	Healthy older adults	Older adults with two doses of the vaccine	3 months	ICS Flow cytometry	High-dimensional flow cytometry	CIR
Fukushima et al. 2025	-	mRNA	Healthy older adults	Older adults with two doses of the vaccine	3 to 6 months	QuantiFERON	SARS-CoV-2 IgG II Quant	CIR HIR

AIM: Activation-induced marker assay; CIR: cellular immune response; CMIA: chemiluminescent microparticle; COPD: chronic obstructive pulmonary disease; ECLIA: electrochemiluminescence sandwich immunoassay; ELISpot: Enzyme-Linked ImmunoSpot; HIR: humoral immune response; ICS: intracellular cytokine staining; mRNA: messenger ribonucleic acid; RA: rheumatoid arthritis.

Table S3. Analysis of immune responses at the time of evaluation.

Author	Vaccine type	HIR (1-3 months)	HIR (≥ 6 months)	CIR (1-3 months)	CIR (≥ 6 months)
Tut et al.	Pfizer-BioNTech Oxford-AstraZeneca	21 days: higher IgG levels	Not present	21 days: higher IFN-γ levels	Not present
Costa et al.	CoronaVac	28 days: higher IgG levels	1 year: It remains stable	28-48 days: high responses of CD4+ T lymphocytes	1 year: It remains stable and then decreases
Rouers et al.	Pfizer-BioNTech Moderna	28 days: higher IgG levels	Significant reduction	7-28 days: high responses of Tfh cell and CD4+ T lymphocytes	Not present
Chaiwong et al.	CoronaVac Oxford-AstraZeneca	4-12 weeks: high levels of antibodies against wild-type, Alpha, Beta, and Delta variants	> 12 weeks: low levels of antibodies against variants (except for the beta variant)	4 weeks: high responses of CD4+ T lymphocytes	Not present
Dalla et al.	Pfizer-BioNTech	14 days: higher IgG levels	Not present	14 days: high responses of T lymphocytes	Not present
Dudley et al.	Pfizer-BioNTech Moderna	2-6 weeks: low levels Spike specific IgG levels	Not present	2-6 weeks: Low levels Spike specific IFN-γ	Not present
Saiag et al.	Pfizer-BioNTech	3 weeks: higher Spike specific IgG levels	Not present	3 weeks: high IFN-γ and TNF-α levels	Not present
Bredholt et al.	Pfizer–BioNTech Moderna	3-6 weeks: higher levels Spike specific IgG levels	Significant reduction	3-6 weeks: no significant improvement in T cell immunity	Not present
Kometani et al.	Pfizer-BioNTech	3 months: higher levels Spike specific IgG levels	6 months: It remains stable	3 months: Low response Spike-specific cTfh1 cells	6 months: not present
Niyomnaitham et al.	Pfizer-BioNTech Oxford-AstraZeneca	2-4 weeks: higher levels Spike specific IgG levels	Significant reduction	2-4 weeks: high responses of T lymphocytes	Not present

Segato et al.	Pfizer-BioNTech	1 month: levels remain at wild-type, BA.2.86, and JN.1 Omicron subvariants	Not present	1 month: low response of CD4+ T lymphocytes	Not present
Vanda et al.	Pfizer-BioNTech	28 days: low IgG levels	Not present	28 days: low response of CD4+ T lymphocytes	Not present
Fukushima et al.	mRNA	3 months: low IgG levels	6 months: significant reduction	3 months: low IFN-γ levels	6 months: significant reduction

CIR: cellular immune response; HIR: humoral immune response; IFN-γ: interferon-gamma; IgG: Immunoglobulin G; mRNA: messenger ribonucleic acid; Tfh: T follicular helper; TNF-α: tumor necrosis factor-alpha.

Table S4. Continuous variables of humoral and cellular immune response in older adults.

Author/Year	Sample	HIR (IgG Median ± SD) Basal comparator	HIR (IgG Median ± SD) Vaccinates	CIR (IFN-γ, TNF-α Median ± SD) Basal comparator	CIR (IFN-γ, TNF-α Median ± SD) Vaccinated
Tut et al. 2021	23	327 ± 2044.51	3102 ± 100 080.10	14.0 ± 7.5*	418.5 ± 235.0*
Saiag et al. 2023	133	50.77 ± 34.83	97.90 ± 9.2	619.11 ± 598.8	1315.88 ± 995.72*
Niyomnaitham et al. 2024	139	1636.36 ± 672.38	2583.69 ± 713.07	120.35 ± 86.27	150.59 ± 155.70*

*Spot-forming units [SFU] per 10^6 PBMCs. SD: standard deviation.

Figure S1. Forest plot of the meta-analysis of three studies on humoral immune response (random-effects model).

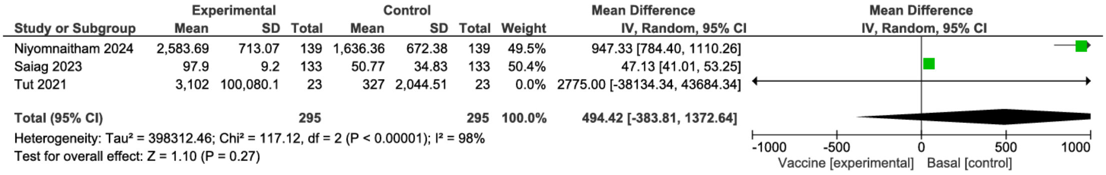

Figure S2. Funnel plot of the meta-analysis on humoral immune response (random-effects model).

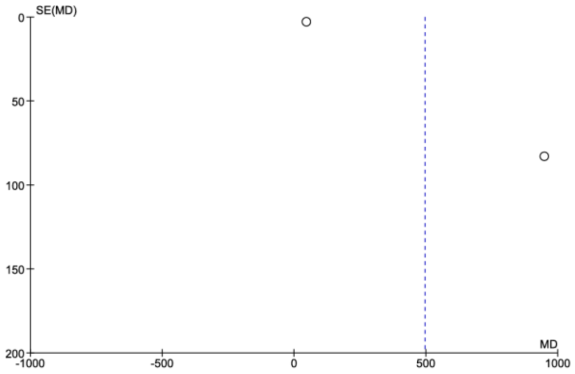

Figure S3. Forest plot of the sensitivity analysis excluding study on humoral response.

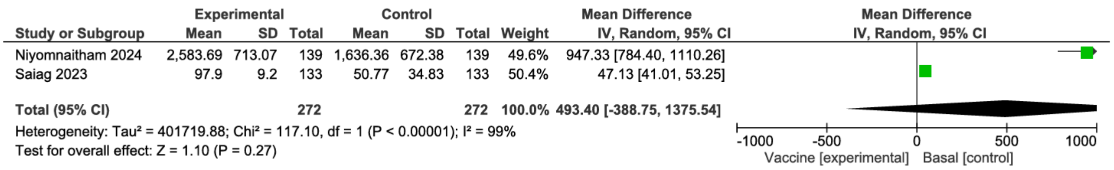

Figure S4. Funnel plot after excluding study in the assessment of humoral response.

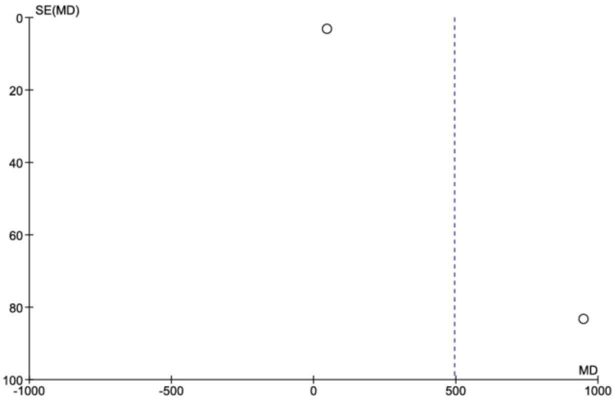

Figure S5. Forest plot of the fixed-effects model analysis for humoral immune response.

Study or Subgroup	Experimental Mean	Experimental SD	Experimental Total	Control Mean	Control SD	Control Total	Weight	Mean Difference IV, Fixed, 95% CI
Niyomnaitham 2024	2,583.69	713.07	139	1,636.36	672.38	139	0.1%	947.33 [784.40, 1110.26]
Saiag 2023	97.9	9.2	133	50.77	34.83	133	99.9%	47.13 [41.01, 53.25]
Tut 2021	3,102	100,080.1	23	327	2,044.51	23	0.0%	2775.00 [-38134.34, 43684.34]
**Total (95% CI)**			**295**			**295**	**100.0%**	**48.40 [42.28, 54.52]**

Heterogeneity: Chi² = 117.12, df = 2 (P < 0.00001); I² = 98%
Test for overall effect: Z = 15.51 (P < 0.00001)

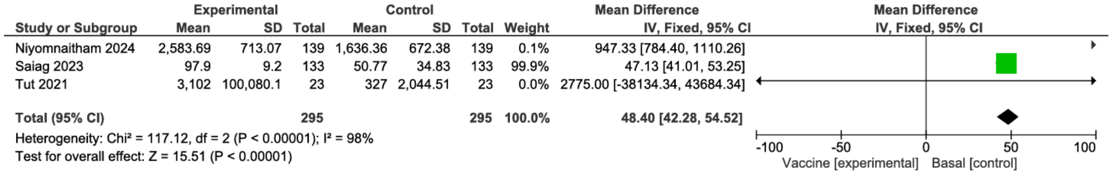

Figure S6. Funnel plot of the meta-analysis using a fixed-effects model for humoral immune response.

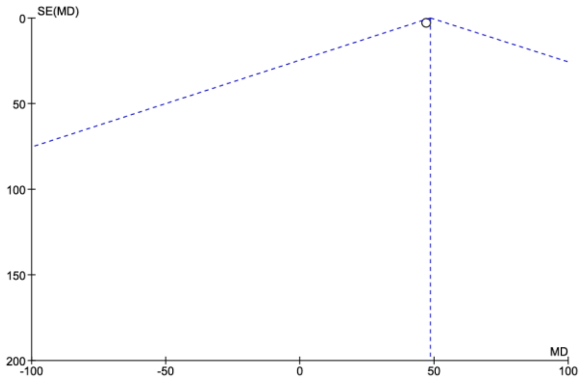

Figure S7. Forest plot of the meta-analysis of three studies on cellular immune response (random-effects model).

Study or Subgroup	Experimental Mean	Experimental SD	Experimental Total	Control Mean	Control SD	Control Total	Weight	Mean Difference IV, Random, 95% CI
Niyomnaitham 2024	150.59	155.7	139	120.35	86.27	139	34.7%	30.24 [0.65, 59.83]
Saiag 2023	1,315.88	995.72	133	619.11	598.8	133	31.4%	696.77 [499.30, 894.24]
Tut 2021	418.5	235	23	14	7.5	23	33.9%	404.50 [308.41, 500.59]
**Total (95% CI)**			**295**			**295**	**100.0%**	**366.60 [8.59, 724.62]**

Heterogeneity: Tau² = 96015.54; Chi² = 92.05, df = 2 (P < 0.00001); I² = 98%
Test for overall effect: Z = 2.01 (P = 0.04)

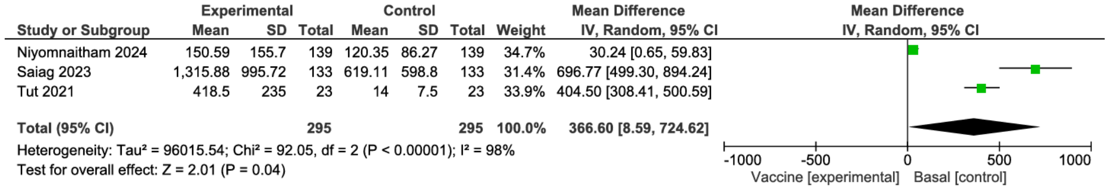

Figure S8. Funnel plot of the meta-analysis on cellular immune response (random-effects model).

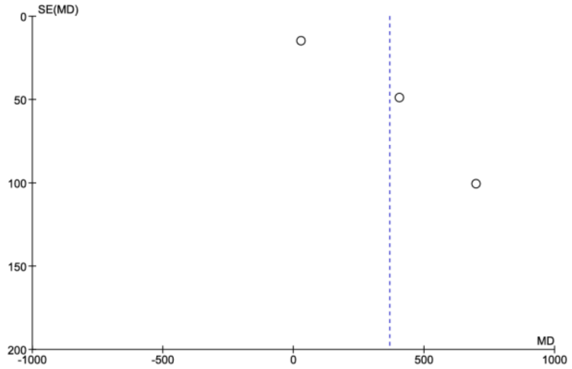

Figure S9. Forest plot of the sensitivity analysis excluding study on cellular response.

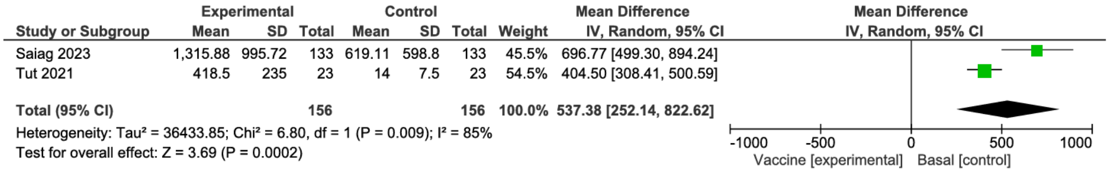

Figure S10. Funnel plot after excluding study in the assessment of cellular response.

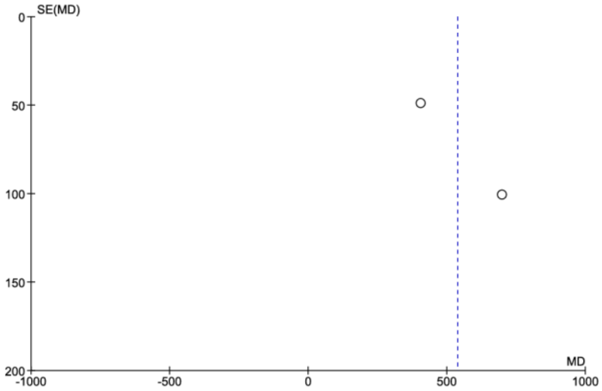

Supplement: Supplementary file 1 [file vaccines-13-00852-s001.zip › vaccines-3763774-supplementary.pdf]
